# Supplementary figures and images for: Bat Dicer antiviral role and subcellular localization differ upon alphavirus infection in two distinct species
Source: PLoS Pathog. 2025 Dec 22;21(12):e1013815. doi: 10.1371/journal.ppat.1013815 (PMC12753048; doi:10.1371/journal.ppat.1013815)

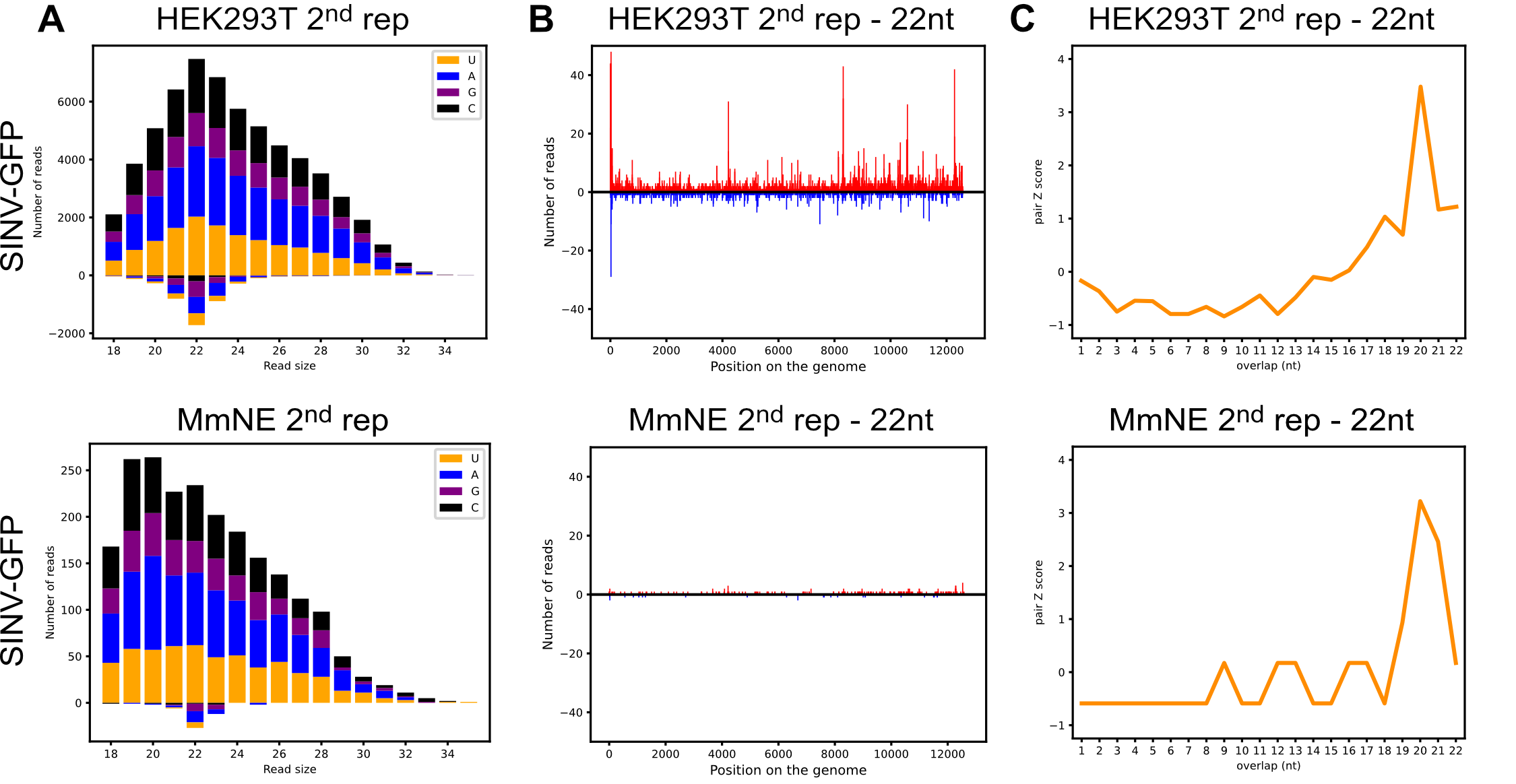

Supplement: S1 Fig — HEK293T or MmNE cells were mock infected or infected with SINV-GFP, then small RNAs were extracted and sequenced. n = 2 independent experiments, see Fig 1 for data from the other replicate. (A) Number of reads that align to the SINV-GFP genome for each cell type based on the read size. The total number of reads is further broken down into colors based on the identity of the first nucleotide of the read: yellow for U, blue for A, purple for G and black for C. (B) Location of the 22 nucleotide (nt) reads along the SINV-GFP genome for each sample. The number of reads falling on the same nucleotide is represented in red if the reads align to the genome (+ strand) and in blue if they align to the anti-genome (- strand). (C) For each sample, 22 nt small RNA pairs that overlap on + and - strands were analyzed and associated z-scores [37] were plotted for the indicated nucleotide overlaps. (TIFF) [file ppat.1013815.s001.tiff]

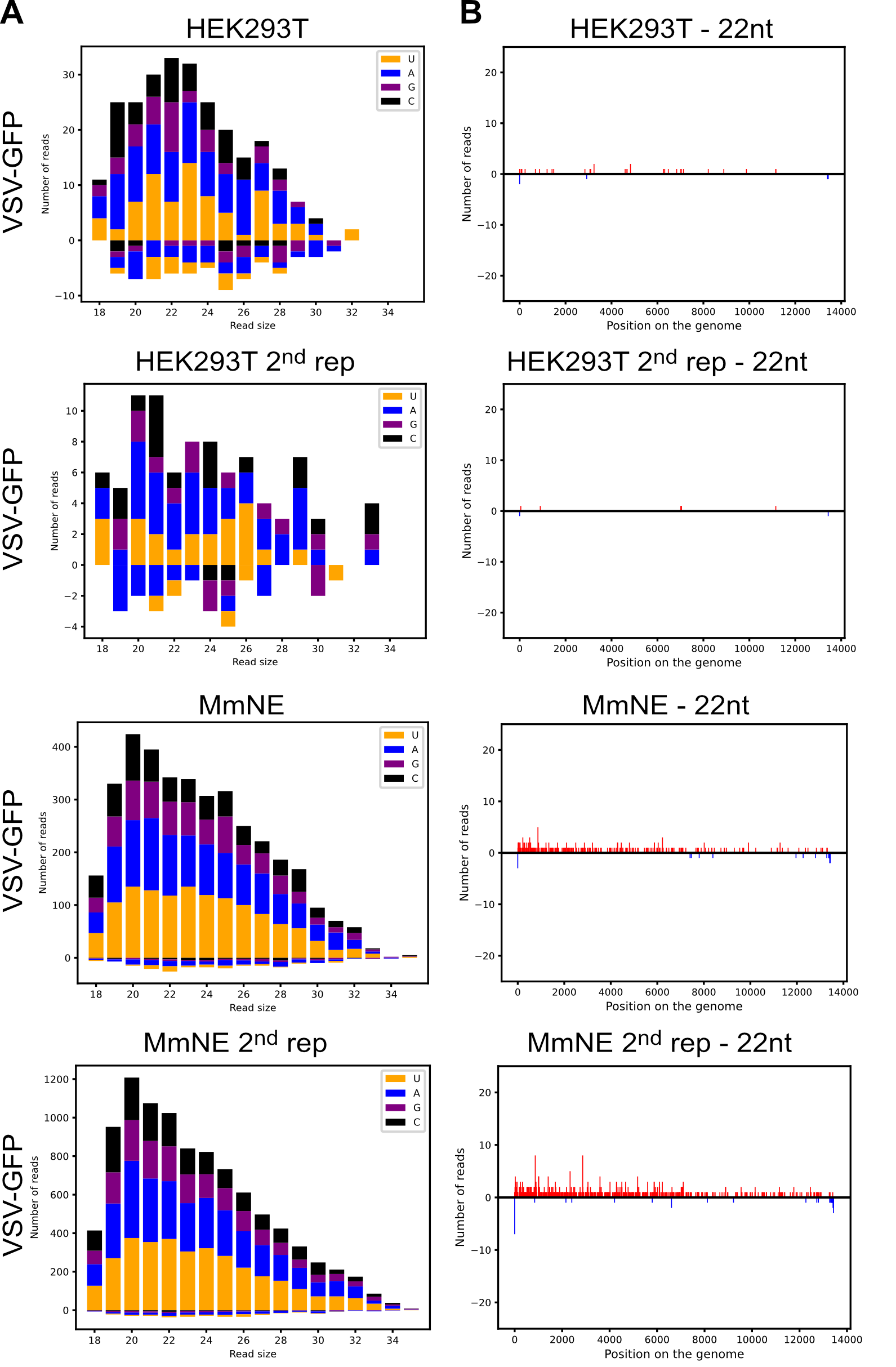

Supplement: S2 Fig — HEK293T or MmNE cells were mock infected or infected with VSV-GFP, then small RNAs were extracted and sequenced. n = 2 independent experiments. (A) Number of reads that align to the VSV-GFP genome for each cell type based on the read size. The total number of reads is further broken down into colors based on the identity of the first nucleotide of the read: yellow for U, blue for A, purple for G and black for C. (B) Location of the 22 nucleotide (nt) reads along the VSV-GFP genome for each sample. The number of reads falling on the same nucleotide is represented in red if the reads align to the genome (+ strand) and in blue if they align to the anti-genome (- strand). For each sample, the number of 22 nt small RNA pairs that overlap on + and - strands was so small (2 and 0 for the HEK293T replicates, 6 and 29 for the MmNE replicates) that the associated Z scores [37] were not plotted. (TIFF) [file ppat.1013815.s002.tiff]

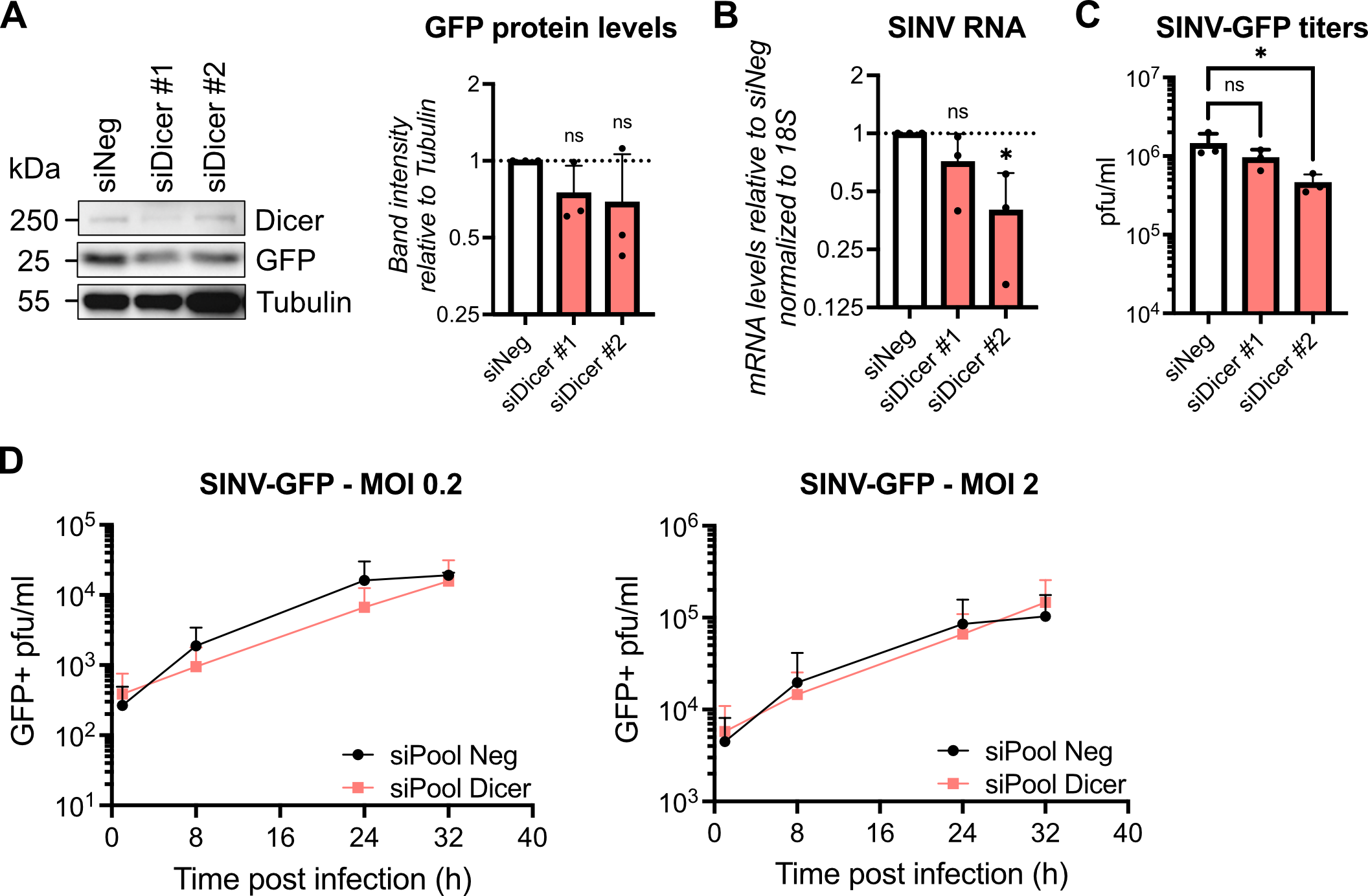

Supplement: S3 Fig — (A-C) MmNE cells treated with two different siRNAs targeting Dicer (siDicer) or a non-targeting control (siNeg) were infected with SINV-GFP for 24h at MOI 0.2. (A) Protein lysates were collected and analyzed by western blot using antibodies for Dicer and GFP. α-Tubulin was used as loading control. Images are representative of 3 independent experiments. GFP band intensities relative to Tubulin band intensities were quantified with ImageJ for each experiment, and means + standard deviations were plotted relative to siNeg infected samples. (B) RNAs were purified and levels of SINV-GFP RNA were quantified by qRT-PCR. Means + standard deviations were plotted relative to siNeg infected samples for 3 independent experiments. (C) Supernatants were collected and viral titers were quantified by plaque assay. Means + standard deviations were plotted for 3 independent experiments. For the RNA and protein graphs, p values were calculated using one sample t tests compared to 1. For the viral titer graph, p values were calculated using an ordinary one-way ANOVA with Dunnett’s multiple comparison test with a single pooled variance compared to siNeg. * p < 0.05; ns: not significant. (D) MmNE cells treated with siPool siRNAs targeting Dicer (siPool Dicer) or a non-targeting control (siPool Neg) were infected with SINV-GFP at MOI 0.2 (left) or 2 (right) for 1h, then the inoculum was removed and replaced with fresh media. Supernatants were collected at this time and at 8h, 24h and 32h post infection. Viral titers were quantified by plaque assay. Means + standard deviations were plotted for 3 independent experiments. (TIFF) [file ppat.1013815.s003.tiff]

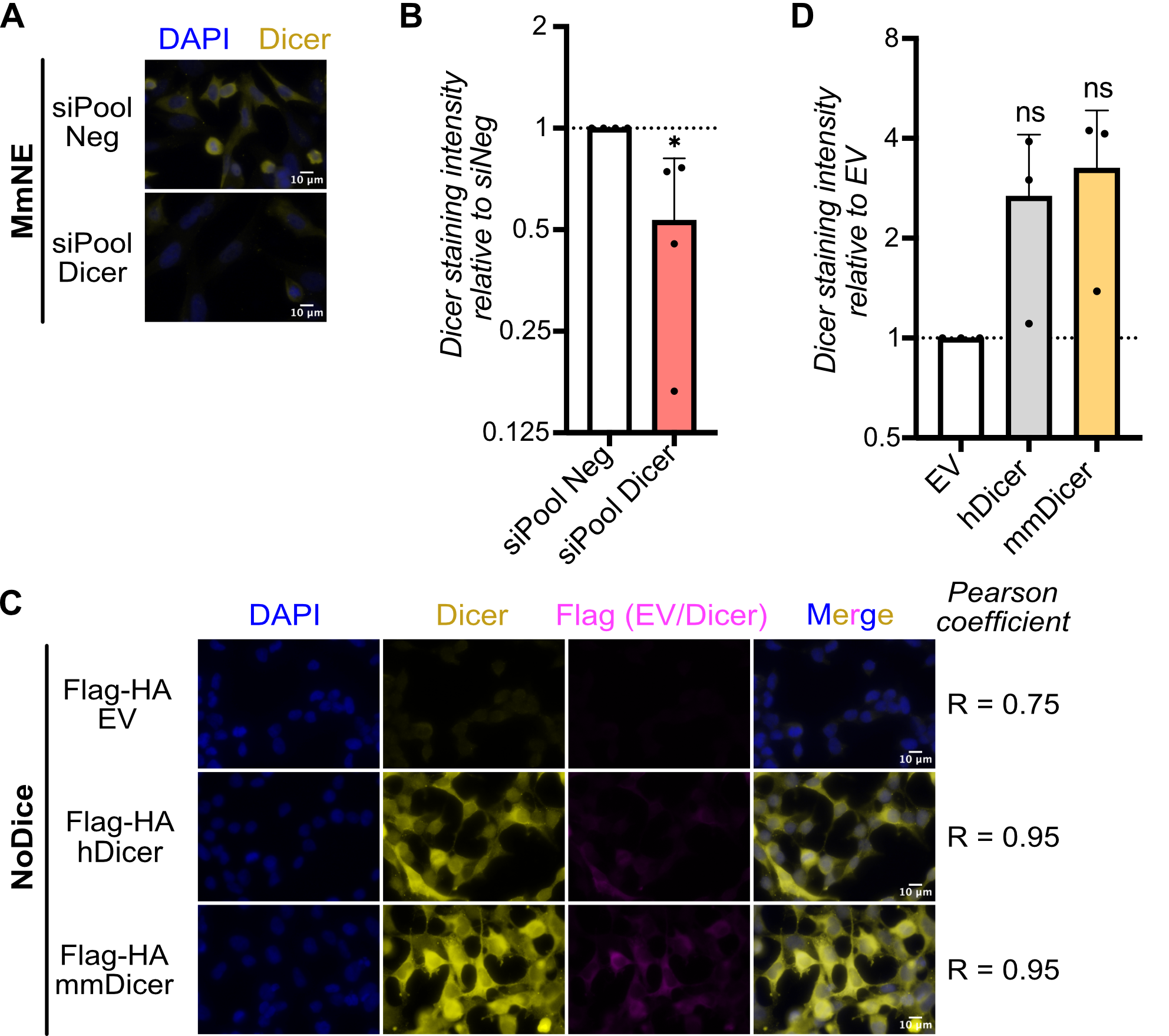

Supplement: S4 Fig — (A-B) MmNE cells treated with siPool siRNAs targeting Dicer (siPool Dicer) or a non-targeting control (siPool Neg) were fixed 48 hours after the last transfection. (A) Dicer staining (yellow) was imaged by epifluorescence microscopy. DAPI staining (blue) indicates cell nuclei. Images are representative of 4 independent experiments. Scale bars 10µm. (B) For each experiment, Dicer staining intensity was quantified and averaged for 3 photos per condition, then normalized to the siPool Neg condition. Means + standard deviations were plotted for all 4 independent experiments. (C-D) HEK293T NoDice cells expressing Flag-HA tagged hDicer, mmDicer or an empty vector control were stained for Dicer (yellow) and Flag (magenta) and imaged by epifluorescence microscopy. DAPI staining (blue) indicates cell nuclei. Images are representative of 3 independent experiments. Scale bars 10µm. Co-localization between Dicer and Flag signals was quantified for the representative images and resulting Pearson correlation R coefficients are indicated on the right. (D) For each experiment, Dicer staining intensity was quantified and averaged for 3 photos per condition, then normalized to the NoDice EV condition. Means + standard deviations were plotted for all 3 independent experiments. For all graphs, p values were calculated using one sample t tests compared to 1. * p < 0.05; ns: not significant. (TIFF) [file ppat.1013815.s004.tiff]

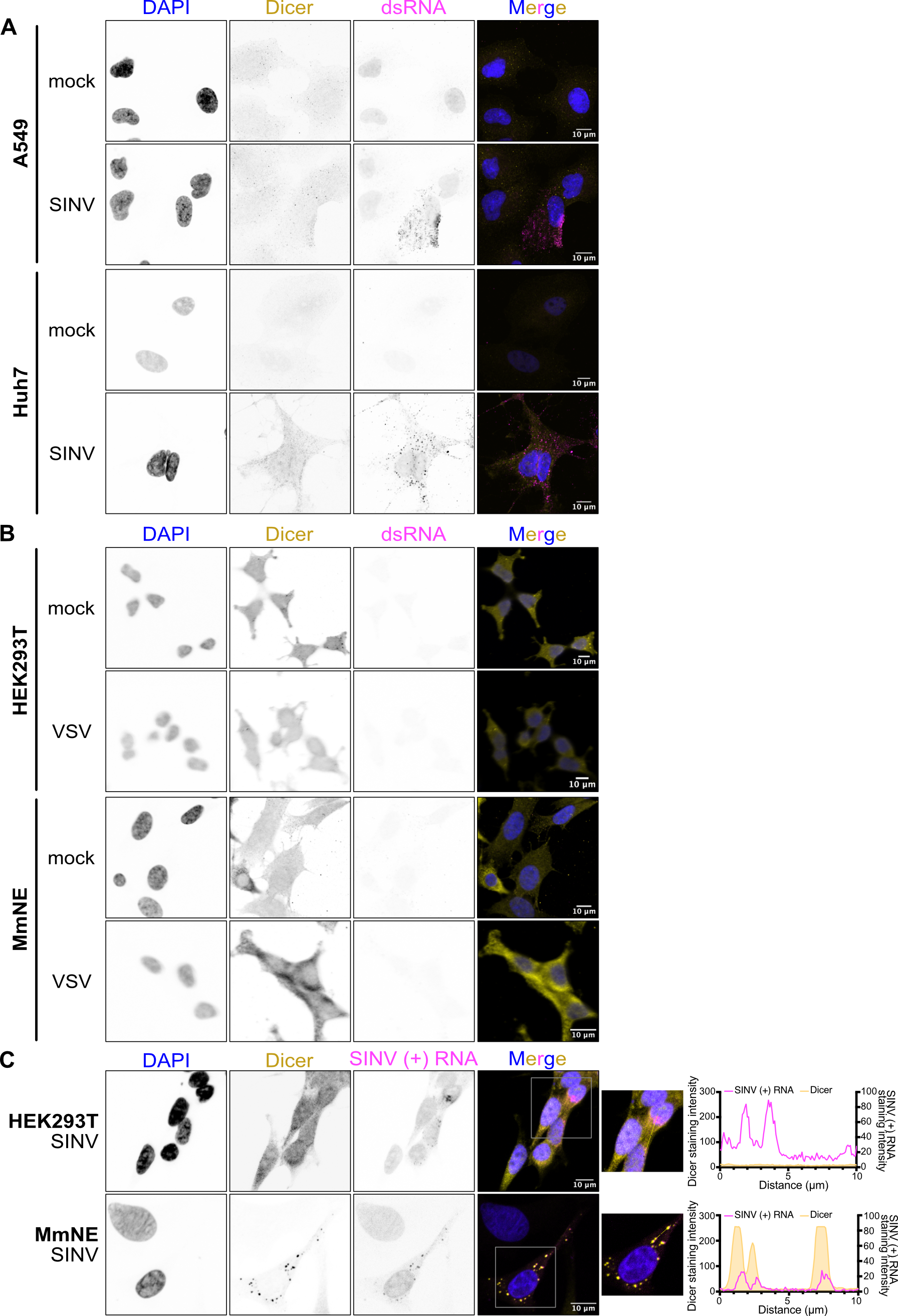

Supplement: S5 Fig — (A) Human A549 and Huh7 cells were mock infected or infected with WT SINV at 24h at MOI 0.2 and 0.02, respectively. Localization of Dicer (yellow) and dsRNA via J2 antibodies (magenta) were imaged by immunofluorescence and confocal microscopy. DAPI staining (blue) indicates cell nuclei. Images are representative of 2 independent experiments. Scale bars 10µm. (B) HEK293T or MmNE cells were infected with WT VSV for 8h at MOI 1. Localization of Dicer (yellow) and dsRNA via J2 antibodies (magenta) were imaged by immunofluorescence and confocal microscopy. DAPI staining (blue) indicates cell nuclei. Images are representative of 2 independent experiments. Scale bars 10µm. (C) HEK293T or MmNE cells were infected with WT SINV for 24h at MOI 0.02 or 4, respectively. Localization of Dicer (yellow) by immunofluorescence and of SINV plus strand (+) RNA (magenta) by FISH was imaged by confocal microscopy. See Fig 4C for epifluorescence microscopy images of the same samples. DAPI staining (blue) indicates cell nuclei. Images are representative of 2 independent experiments. Scale bars 10µm. A zoomed-in image of the boxed area in the merge images was added. The graphs show the signals for each channel along the line drawn in the zoomed in images. The staining intensity of SINV plus strand (+) RNA was too low on confocal images to calculate accurate Pearson correlation R coefficients. (TIFF) [file ppat.1013815.s005.tiff]

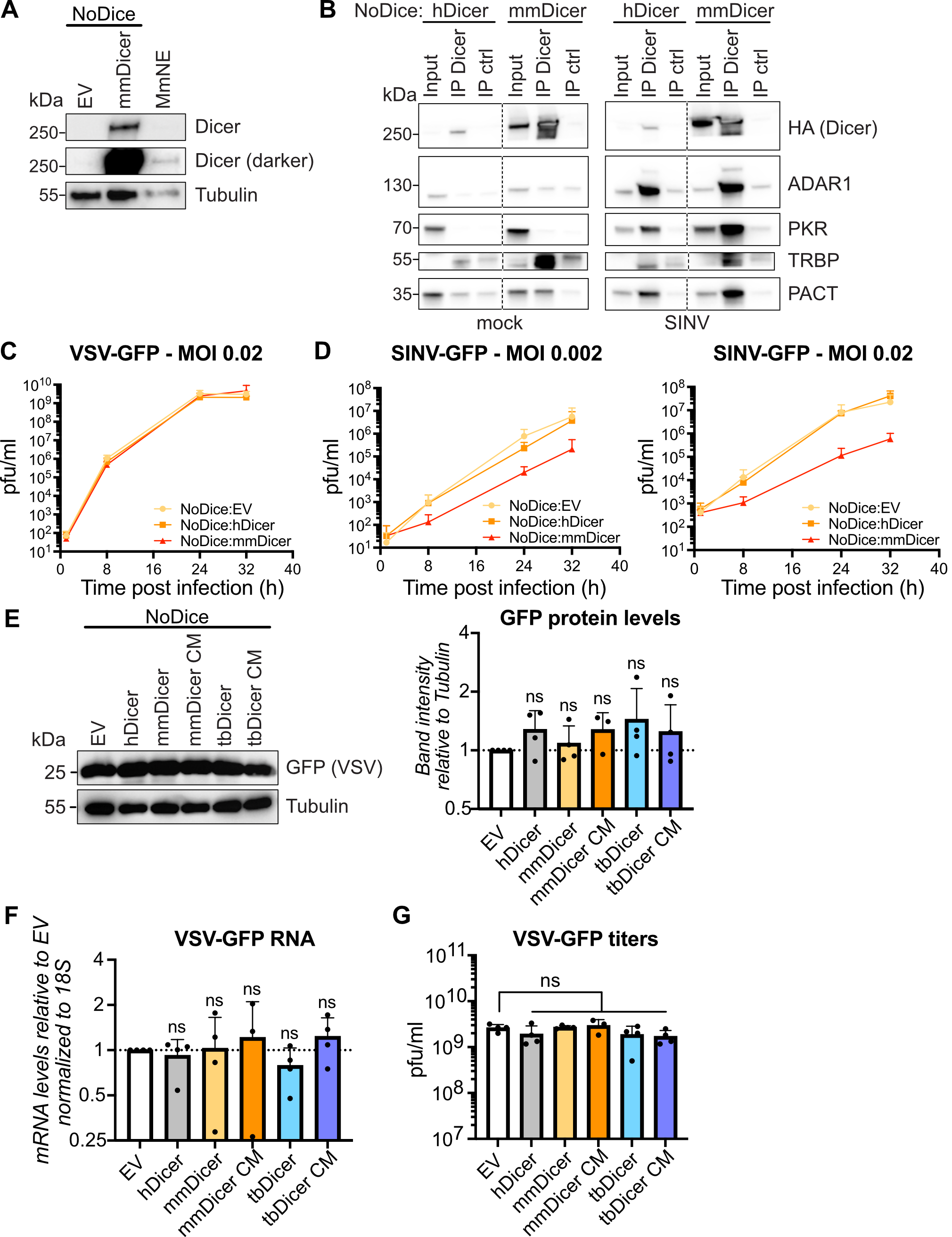

Supplement: S6 Fig — (A) Expression of Dicer was analyzed by western blot for MmNE cells or HEK293T NoDice cells expressing Flag-HA-tagged mmDicer or an empty vector control. α-Tubulin was used as loading control. (B) HEK293T NoDice cells expressing Flag-HA-tagged hDicer or mmDicer were mock infected or infected with SINV-GFP for 24h at MOI 0.02. Cells were then lysed, a small aliquot was kept as input, and Dicer was immunoprecipitated with anti-HA coated magnetic beads (IP Dicer) or anti-myc coated beads (IP ctrl). Eluted proteins were analyzed by western blot using antibodies for the known hDicer interactors ADAR1, PKR, TRBP and PACT. Anti-HA antibodies show efficient immunoprecipitation of hDicer and mmDicer. Images representative of 2 independent experiments. (C-D) HEK293T NoDice cells expressing Flag-HA-tagged hDicer, mmDicer or an empty vector control were infected with (C) VSV-GFP at MOI 0.02 or (D) SINV-GFP at MOI 0.002 (left) or 0.02 (right) for 1h, then the inoculum was removed and replaced with fresh media. Supernatants were collected at this time and at 8h, 24h and 32h post infection. Viral titers were quantified by plaque assay. Means + standard deviations were plotted for 3 independent experiments. (E-G) HEK293T NoDice cells expressing Flag-HA tagged hDicer, mmDicer, mmDicer CM, tbDicer, tbDicer CM or an empty vector control were infected with VSV-GFP at MOI 0.02 for 24h. (E) Protein lysates were collected and analyzed by western blot using antibodies for GFP. α-Tubulin was used as loading control. Images are representative of 3 (mmDicer CM) or 4 independent experiments. GFP band intensities relative to Tubulin band intensities were quantified with ImageJ and the mean + standard deviation was plotted relative to empty vector control for each experiment. (F) RNAs were purified and levels of VSV-GFP RNA were quantified by qRT-PCR. Means + standard deviation normalized to 18S were plotted relative to empty vector control for 3 (mmDicer CM) or 4 independent experiments. (G) Su [file ppat.1013815.s006.tiff]

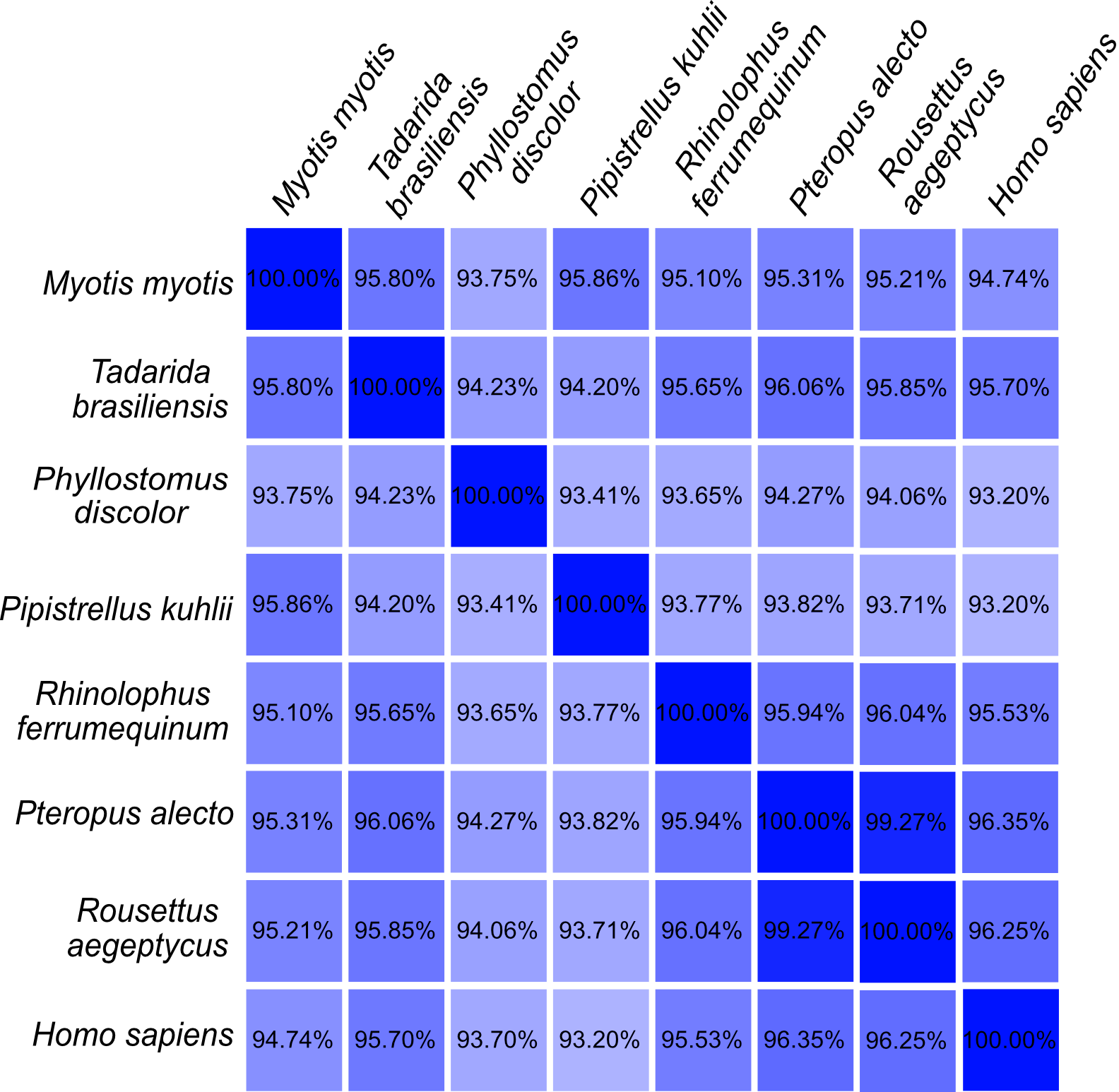

Supplement: S7 Fig — Percentage homology was calculated for each Dicer amino acid sequence from the indicated species and represented as a similarity matrix. (TIFF) [file ppat.1013815.s007.tiff]
