# Supplementary material for: Bat Dicer antiviral role and subcellular localization differ upon alphavirus infection in two distinct species
Source: PLoS Pathog. 2025 Dec 22;21(12):e1013815. doi: 10.1371/journal.ppat.1013815 (PMC12753048; doi:10.1371/journal.ppat.1013815)
Supplement: Attachment — Submitted filename: 159758_1_rebuttal_3488372_t1lb83.pdf [file ppat.1013815.s008.pdf]

Revision Plan

Review COMMONS

Manuscript number: RC-2025-03114
Corresponding author(s): Sébastien Pfeffer

1. General Statements

We would like to thank the reviewers for their overall positive evaluations of our manuscript and for their invaluable suggestions that will allow us to reinforce our conclusions. We acknowledge that there is some work to be done and are ready to address most of the reviewers' comments as detailed in our replies below.

2. Description of the planned revisions

Reviewer 1

1. The findings that mmDicer is proviral in bat cells relies exclusively on the observation that the depletion of Dicer in M. myotis cells leads to a reduced accumulation of SFV and SINV at the RNA and protein levels (figure 2). Heterologous expression of mmDicer in HEK 293T NoDice doesn't lead to an increase permissivity to viral infections (figure 1) and the accumulation of Dicer foci is only observed in M. myotis cells but not when mmDicer is expressed in HEK 293 NoDice cells (figure 6). Given that the key finding of this manuscript relies on these knockdown experiments, the authors should ensure that the impact on viral infections is due to the specific silencing of mmDicer and not caused by off-target effects of their siRNA-mediated approach. The authors designed a siRNA pool to efficiently knock-down mmDicer. They should validate their findings by using individual Dicer siRNA and verify whether the decrease SFV/SINV accumulation is observed with at least two individual siRNAs targeting Dicer. It would also strengthen their findings if they could show a complementation experiment in which a mmDicer (designed to not be affected by the siRNA-mediated silencing) is introduced exogenously in Dicer-depleted cells and show that it rescues the observed decrease in viral accumulation to demonstrate that the proviral role is strictly dependent on mmDicer. Alternatively, the authors could consider a CRISPR/Cas9 genome editing approach to knockout Dicer in bat cells to test whether this proviral effect is confirmed.

> Reply: *We agree with this reviewer that it is important to provide evidence for the specificity of the knock-down and to rule out any off-target effect of the siRNAs. This is the reason for using the siTool technology, which relies on the use of a pool of 30 siRNAs that are transfected at a final concentration of 3 nM. This means that each individual siRNA in the pool is at a concentration of 0.1 nM, so the possibility of off-target effect is largely avoided and the efficiency of silencing is boosted by the cooperative activity of many siRNAs (see https://www.sitoolsbiotech.com/documents/sipools/siPOOLBrochure2019_Web.pdf for more details). This being said, we agree that it would be better to confirm that the observed effect can be recapitulated using a single siRNA and that a*

complementation experiment would definitely strengthen our findings. For this reason, we will test two individual siRNAs targeting the 3' UTR of mmDicer, which will allow us to complement the knock-down by transfecting a cDNA construct. Regarding the CRISPR/Cas9 genome editing approach, we will give it a try, but Dicer is notoriously difficult to knock-out, so we cannot be sure that this will be successful.

2. Figure 2: the authors knock-downed Dicer in M. myotis nasal epithelial cells and carried out infections with SINV-GFP and SFV. The authors conclude that Dicer is proviral as its depletion causes a decrease in SINV-GFP and SFV accumulation. While this conclusion is supported by the decrease levels of viral RNA and protein levels upon Dicer depletion (figure 2D, 2E, 2G), the effect on the viral titers is non-significant for both viruses (Figure 2C and 2F) based on the statistical analysis. This reviewer appreciates that the titers are lower upon Dicer knockdown, which support the authors' findings at the viral RNA and protein levels. However, as these results are central to the core message of the manuscript, the authors should provide evidence that this proviral effect observed is statistically significant on viral titers by perhaps providing additional repeats and/or comment on this observation.

Reply: Indeed, we agree that even if the effect of Dicer knockdown results in a lowering of the viral titer, it would be better to have a statistically significant effect. We will repeat the experiment to increase the number of replicates and the power of the statistical test.

3. a) In figure 4 and 5, the authors nicely show that mmDicer accumulate to cytoplasmic foci in M. myotis cells upon infection with SFV and SINV and these foci co-localise with double-stranded RNA. The authors used a commercial polyclonal antibody against Dicer (A301-937A, Bethyl according to the Material and Methods section) which is specific to human Dicer to carry out their immunostaining in bat cells. The authors should provide evidence that this antibody indeed recognises/crossreacts with mmDicer as well and that the staining shown is indeed specific to mmDicer localisation especially because the heterologous expression of HA-tagged version of mmDicer in HEK 293T NoDice cells did not show this accumulation of cytoplasmic foci. The authors should verify the specificity of their mmDicer immunostaining by performing the same labelling in bat cells in which Dicer is knock-downed (or knock out) by individual and validated siRNA against mmDicer. The decrease signal of bat Dicer staining using the anti-human Dicer antibody would indicate specificity.

Reply: the reviewer is correct in its assertion and it is important to provide evidence that the protein that is detected by the anti-human Dicer antibody in bat cells is indeed Dicer. We will perform the suggested experiment and do an immunostaining using the Dicer antibody in bat cells upon Dicer knockdown.

3. b) Another complementary approach would be to test their Dicer staining between HEK NoDice cells (no Dicer present) versus NoDice complemented with either mmDicer or human Dicer

constructs, which would then indicate how much the anti-human Dicer antibody recognises bat Dicer.

> <u>Reply:</u> *this complementary approach should yield even cleaner result than the previous one as there will be no expression of Dicer at all in the HEK NoDice cells. Therefore, we should be able to measure the increase of signal in the IF upon expression of either human or bat Dicer. We will perform this experiment together with the other one suggested above. In addition, since the constructs are tagged, we might be able to do a double-staining and verify the colocalization of the two signals.*

3. c) In addition, the authors should overexpress HA-tagged mmDicer in M. myotis nasal epithelial cells and test whether HA-mmDicer accumulate into foci upon infection using an anti-HA immunostaining. This would confirm that these accumulation into foci indeed is specific to mmDicer but also would reinforce the authors' findings that host factors within bat cells are important for this formation into foci since mmDicer expression in HEK 293T No Dice cells didn't show this phenotype upon infection (figure 6). OPTIONAL: it would be interesting to overexpress HA-tagged human Dicer into M. myotis nasal epithelial cells as well to then test using anti-HA staining whether human Dicer in presence of host factors from the bat can accumulate into cytoplasmic foci or not upon viral infection.

> <u>Reply:</u> *we could perform the suggested experiment, but we might face the issue that transfected cells might mount an immune response, which makes them resistant to the infection. We have observed indeed that we needed to use a higher MOI to infect cells after they have been transfected. Since we will have controls in place, this might not be too much of a problem, but we will have to keep it in mind. Alternatively, we will perform a lentiviral transduction of the cells.*

4. This reviewer appreciates that this might be judged as beyond the scope of this study since it is focused on the role of Dicer in M. myotis. However, the observation that mmDicer accumulates into foci containing as well viral dsRNA is very interesting and it would significantly improve the manuscript if the authors would provide further indications that this phenotype is related to the lack of antiviral activity of mmDicer compared to what has been previously shown in other bat species (P.alecto and T. brasiliensis). In other words, is this accumulation of mmDicer into foci responsible for its different impact on virus infection? It would therefore be insightful to compare Dicer localisation upon infection in M. myotis versus P.alecto and/or T. brasiliensis bat cells in which Dicer was shown to be antiviral and test whether this accumulation in foci is only observed in bat cells in which Dicer is proviral (M. myotis) but not in the other bat cells in which Dicer is antiviral (P.alecto and/or T. brasiliensis).

> <u>Reply:</u> *this is something that we have been wondering about and we have therefore started to look for the cell lines that have been described in the two published studies. While it proved difficult to find the PaKi cells from P. alecto bats, which is*

not commercially available, we have obtained the Tblu cells from T. brasiliensis and will look at Dicer localization in this model. However, we have to pay attention to the fact that the published data reported a contribution of RNAi in this cell line upon SARS-CoV-2 infection and that we will be using SINV. In addition, we do not know yet whether the anti-Dicer antibody will cross react with the T. brasiliensis Dicer protein.

5. OPTIONAL: Given the difference between the provial role of mmDicer compared to the antiviral activity of Dicer in cells from P.alecto and T. brasiliensis bat cells, it would strengthen the authors' findings. if additional experiments would be conducted in parallel using M. myotis, P.alecto and/or T. brasiliensis cells. Notably knocking down Dicer in both M. myotis, P.alecto and/or T. brasiliensis cells, compare the impact on viral infections with SINV, SFV, VSV and correlate any observed difference in phenotype with putative variations in the formation of foci.

Reply: it would indeed be really nice to be able to do the Dicer knockdown experiment in several bat cell lines and to correlate the phenotype with the formation of foci. This experiment might take a long time and we are not sure to be able to realize it in a reasonable amount of time. It could however be the subject of another manuscript further down the line.

Minor comments

• Figure 2I: The authors performed a knockdown of Dicer in M. myotis nasal epithelial cells and monitor the impact on VSV-GFP infection. They found that knocking down Dicer leads to an increase in GFP protein and RNA levels suggesting an antiviral role of Dicer while, in contrast, no effect is observed on the production of infectious particles (figure 2H). On the western blot there is only a slight/weak increase of GFP protein level observed upon Dicer knockdown. Yet, the quantification of the band intensity shows a 4-fold increase relative to tubulin and compared to cells treated with siRNA control. This 4-fold increase seems exaggerated given the low increase in the intensity shown on the blot. This discrepancy is most likely due to the lower intensity of tubulin in the western blot analysis of siDicer-treated cells compared to siNeg-treated cells. The authors should reload their western blot with equal amount of protein extract loaded to ensure that the results shown on the western blot are in line with the quantification.

Reply: the signal quantification for this experiment was done across several replicates, but we agree that the observed effect seems exaggerated when compared to the signal seen on the blot. We observed important variations between replicates, but we will make sure that this was not due to a problem in the analysis and reload the western blot if needed.

• Figure 3D: the authors mention that in both HEK293T cells and M. myotis nasal epithelial cells infected with SINV-GFP, there was an enrichment of 22-nucleotides (nt) paired positive and negative sense reads that overlapped with a 2-nt overhang, typical of Dicer cleavage. In Figure 3D, the data shows indeed that the duplexes are enriched for reads of 22-nt but it is unclear how

this analysis reveals a 3' 2nt overhang within these duplexes. Can the authors clarify this point and if the data provided in that particular analysis indeed doesn't allow to detect these overhangs, please rephrase accordingly or provide additional analysis to support that point.

> Reply: *In Figure 3D, the graphs show the probability of pairing of all 22 nucleotides sequence mapping either to the plus or the minus strand of the viral RNA. Thus, for each sequence mapping to the plus strand, the number of sequences mapping to the minus strand with a full or partial overall is counted. A corresponding probability of pairing and Z score is calculated for each number of overlapping nucleotides (for more information on the calculation see Antoniewski (2014) Computing siRNA and piRNA Overlap Signatures. In Animal Endo-SiRNAs: Methods and Protocols, Werner A (ed) pp 135–146. New York, NY: Springer). The Z score peaks for an overlap of 20 nt in both HEK293T and M. myotis nasal epithelial cells infected with SINV. This means that there is a higher probability of two 22 nt sequence to pair along 20 nt, and thus that there are two unpaired nucleotides at the extremities of the duplexes. This higher Z score at 20 nt is not seen in VSV-infected cells. We will rephrase the text in the manuscript to make this point clearer.*

• Typo: page 5, line 152: the authors mention that Dicer knock down had an antiviral effect against VSV-GFP infection at the RNA and protein levels. However, the data in Figure 2I and 2J show an increase in both GFP RNA and proteins levels upon knockdown of Dicer. Although this data suggests that Dicer is antiviral against VSV, the knockdown of Dicer itself is not antiviral but rather proviral/increase virus accumulation. Please rephrase this sentence to avoid confusions.

> Reply: *thank you for spotting this typo. We have corrected it accordingly.*

Reviewer 2.

1. Figure 1 relies on transduction of cells and antibiotic selection to obtain mmDicer-expressing cells. Although we would expect that every cell expresses the construct of interest, this is not always the case, depending on the cell type and toxicity of the construct. As the constructs are tagged, I suggest that the authors use flow cytometry to measure expression levels in a single cell manner. While doing so, they can infect with SINV-GFP and correlate GFP signal with construct expression in each cell, providing a more accurate measurement of mmDicer effect on viral infection. Alternatively, the authors could use live microscopy, as done in Fig 2, to obtain similar data.

> Reply: *the reviewer is correct that we did not go for monoclonal selection of our mmDicer-expressing cells and therefore that there could be some cell-to-cell variation in expression. However, we have done immunostaining of Dicer in these cells and did not see drastic differences in expression, so we do not think this*

should impact SINV-GFP expression in a major way. We will provide these images and a quantification of the Dicer signal as a supplementary figure.

2. For Fig 1C and 1F, it would be great to have growth curves with two different MOIs, instead of a single time point, to ensure that a putative antiviral effect is not missed. Same goes for Fig 2C, especially when the authors document quite a big defect on GFP expression (a proxy for SINV infection) when Dicer is knocked down (Fig 2B). There may be a bigger difference in titers at earlier time points. This matter runs throughout the manuscript. I do not suggest that the authors should provide growth curves every time viral titers are measured, but it is still worth doing it for the 2-3 key experiments of the paper.

Reply: we will perform growth curves of virus infection for the key experiments in the manuscript as suggested. We already have done kinetic measurements of GFP accumulation at different MOIs, which we can provide as supplementary data, but we agree with the reviewer that GFP signal should not been used as the only proxy for the infection and that measuring viral titers by plaque assay is important as well.

3. Figure 4, could the authors provide a proof that the Dicer antibody is specific in the bat context? This can be done by staining Dicer in bat cells knocked down for Dicer and infected with SINV. The apparition of foci upon anti-Dicer antibody staining should be abbrogated or severely impaired by the knock-down.

Reply: see our reply to point 3 of Reviewer 1.

4. Fig 5C, please provide a quantification of the images.

Reply: these microscopy images have not been quantified because they have been obtained with an epifluorescence microscope. Indeed, the Pearson correlation coefficient can only be obtained using a confocal microscope. In fact, we have tried to use a confocal microscope to take pictures of these FISH images, but the SINV gRNA signal was too weak or the dots too small to be properly visualized. Furthermore, there is a very large difference in signal intensity between HEK293T and M. myotis cells, making it difficult to define a signal threshold compatible for both cell lines.

5. l.263, when comparing this work with the recent publications on bat antiviral RNAi, the authors could also provide the percentage identity between Dicers from different species.

Reply: this is a valid point, we have looked at the percentage identity between Dicer proteins from different bat species but we did not include this in our manuscript. We will provide this analysis in the revised version together with a comparison of Dicer from other mammals as a reference point.

Reviewer 3.

1. Without direct comparison to the other bat species Dicers (especially where RNAi activity has been suggested as antiviral in previous publications) there is little in this paper that can be concluded about global aspects of bat dicer/RNAi.

> *Reply: see our reply to point 4 of Reviewer 1. We are planning to look at least in Tblu cells whether there is also a relocalization of Dicer upon SINV infection. So far, we could not obtain PaKi cells, but we are still looking and should we get those, we will test them as well.*

Minor

2. What rules out that the mmDicer re-localization observed in the immortalized mm nasal epithelial is due simply to greater expression levels over the NoDice cells heterologously expressing mmDicer?

> *Reply: we will provide an immunoblot to show the level of Dicer expression between HEK NoDice + mmDicer and M. myotis nasal epithelial cells as suggested below to address this point.*

3. Although partially addressed in the text stating the generally long half-life of miRNAs, it seems the simplest explanation for this observation is due to some activity of a shorter-lived miRNA is required for optimal alphavirus replication is the mm nasal epithelial cells.

> *Reply: this is an interesting hypothesis that would prove difficult to test in a reasonable amount of time. We thank the reviewer and will mention this possibility in the discussion of the revised manuscript.*

Suggestions that could enhance the magnitude of conclusions that can be drawn from this work.
Major

• Making NoDice cells expressing other bat species Dicers, including those with claims that RNAi is antiviral, would address how universal these current observations are to bats/cell lines.

> *Reply: this could be an alternative to the use of P. alecto or T. brasiliensis cell lines that we have mentioned above. We will try to clone Dicer from the Tblu cells that we have in the laboratory. Since we do not have PaKi cells at the moment, it will be more complicated for the Pteropus Dicer, but one possibility could be to synthesize it. However, Dicer is a big gene so it could prove tricky.*

• Including an immunoblot showing that mm cells express mmDicer no more abundantly than the heterologous NoDice cells would allow ruling out the trivial explanation that foci occur at a certain critical mass of Dicer

> *Reply: yes, we will provide this piece of data as stated in reply to point 2.*

Minor
• I believe line 151 " In contrast, Dicer
knock down had an ANTIVIRAL effect against VSV-GFP infection at the RNA and protein levels, but no difference in titers was found (Fig. 2H-J)." should be " In contrast, Dicer knock down had an PROVIRAL effect against VSV-GFP infection at the RNA and protein levels, but no difference in titers was found (Fig. 2H-J)."

> *Reply: thank you for spotting this error, which was also mentioned by Reviewer 1, we have corrected this in the text.*

3. Description of the revisions that have already been incorporated in the transferred manuscript

We have provided a revised version in which we have only corrected the typo that was spotted by Reviewers 1 and 3 (about the impact of Dicer knock-down on VSV, see above) (page 5, line 152). In addition, we also added the sequences of two primers that we forgot in the previous version (page 15, line 417). All the other revisions will be incorporated in a future version of the manuscript.

4. Description of analyses that authors prefer not to carry out

As stated in our replies to reviewers' comments, we will try to address all of their points. However, at this moment, we are not sure whether we will be able to carry out the experiments in other bat cell lines, especially the PaKi cells that we could not obtain. We will test the Tblu cell line that is currently growing in the laboratory, but we yet have to verify that the Dicer antibody will also cross react with the Dicer of this species. Given its similarity with *Myotis myotis* Dicer, this should not be a problem, but we will not know before we have done the experiment.
The only experiment that we think might not be necessary is the proposed flow cytometry analysis of Dicer expression in the HEK293T NoDice cells transduced with mmDicer. However, we will provide microscopy images of immunostained cells, which should answer this point.
