# Supplementary material for: Bat Dicer antiviral role and subcellular localization differ upon alphavirus infection in two distinct species
Source: PLoS Pathog. 2025 Dec 22;21(12):e1013815. doi: 10.1371/journal.ppat.1013815 (PMC12753048; doi:10.1371/journal.ppat.1013815)
Supplement: Attachment — Submitted filename: gaucherand_rebuttal.pdf [file ppat.1013815.s009.pdf]

Rebuttal letter

We would like to thank the reviewers and the editor for their very pertinent comments, which we have almost entirely addressed as indicated in the replies below. Given the prominent addition of new data, we have reorganized the figures significantly in order to incorporate the new panels and facilitate the comprehension of this revised version. We are listing below the list of changes in figure number between the initial and the revised version of the manuscript.

REVISION	ORIGINAL
-Figure 1	Figure 3
-Figure S1	Part of Figure S1
-Figure S2	Part of Figure 3 and Figure S1
-Figure S3	New data
-Figure 2	Figure 2
-Figure S4	New data
-Figure 3	Figure 4
-Figure 4	Figure 5
-Figure S5	Figure S2 + new data
-Figure S6	Part of Figure 6 + new data
-Figure 5	New data
-Figure 6	Part of Figure 6 + new data
-Figure S7	New data

The changes in the text can be visualized in the document where changes compared to the original version are highlighted. Below are our replies to reviewers' comments.

Replies to reviewers
Reviewer 1
1. The findings that mmDicer is proviral in bat cells relies exclusively on the observation that the depletion of Dicer in M. myotis cells leads to a reduced accumulation of SFV and SINV at the RNA and protein levels (figure 2). Heterologous expression of mmDicer in HEK 293T NoDice doesn't lead to an increase permissivity to viral infections (figure 1) and the accumulation of Dicer foci is only observed in M. myotis cells but not when mmDicer is expressed in HEK 293 NoDice cells (figure 6). Given that the key finding of this manuscript relies on these knockdown experiments, the authors should ensure that the impact on viral infections is due to the specific silencing of mmDicer and not caused by off-target effects of their siRNA-mediated approach. The authors designed a siRNA pool to efficiently knock-down mmDicer. They should validate their findings by using individual Dicer siRNA and verify whether the decrease SFV/SINV accumulation is observed with at least two individual siRNAs targeting Dicer. It would also strengthen their findings if they could show a complementation experiment in which a mmDicer (designed to not be affected by the siRNA-mediated silencing) is introduced exogenously in Dicer-depleted cells and show that it rescues the observed decrease in viral accumulation to demonstrate that the proviral role is strictly dependent on mmDicer. Alternatively, the authors could consider a CRISPR/Cas9 genome editing approach to knockout Dicer in bat cells to test whether this proviral effect is confirmed.

> <u>Reply:</u> *We agree with this reviewer that it is important to provide evidence for the specificity of the knock-down and to rule out any off-target effect of the*

siRNAs. This is the reason for using the siTool technology, which relies on the use of a pool of 30 siRNAs that are transfected at a final concentration of 3 nM. This means that each individual siRNA in the pool is at a concentration of 0.1 nM, so the possibility of off-target effect is largely avoided and the efficiency of silencing is boosted by the cooperative activity of many siRNAs (see https://www.sitoolsbiotech.com/documents/sipools/siPOOLBrochure2019_Web.pdf for more details). This being said, we designed two individual siRNAs targeting mmDicer mRNA and tested their effect on SINV infection. These results are shown on the new Figure S3 and discussed in the text on lines 189-202. Briefly, we confirmed that both siRNAs resulted in a moderate knock down of Dicer at the protein level and had a modest antiviral effect on SINV (see Figure S3A-C). Unfortunately, we did not manage to perform the rescue experiment with these individual siRNAs. One reason is the rather inefficient knock down induced by those and another is due to the fact that it proved difficult to overexpress mmDicer in Myotis myotis nasal epithelial (MmNE) cells, be it by transient transfection or lentiviral transduction. Nonetheless, we believe that our results show that both when using "classical" siRNA transfection or "pooled" siRNAs, knocking down Dicer expression levels does not confer a proviral phenotype, which would have been expected if Dicer had an antiviral role in Myotis myotis cells.
Regarding the CRISPR/Cas9 genome editing approach, we did not manage to obtain knock out clones in the time allotted for revising this manuscript.

2. Figure 2: the authors knock-downed Dicer in M. myotis nasal epithelial cells and carried out infections with SINV-GFP and SFV. The authors conclude that Dicer is proviral as its depletion causes a decrease in SINV-GFP and SFV accumulation. While this conclusion is supported by the decrease levels of viral RNA and protein levels upon Dicer depletion (figure 2D, 2E, 2G), the effect on the viral titers is non-significant for both viruses (Figure 2C and 2F) based on the statistical analysis. This reviewer appreciates that the titers are lower upon Dicer knockdown, which support the authors' findings at the viral RNA and protein levels. However, as these results are central to the core message of the manuscript, the authors should provide evidence that this proviral effect observed is statistically significant on viral titers by perhaps providing additional repeats and/or comment on this observation.

<u>*Reply:*</u> *Indeed, we agree that even if the effect of Dicer knockdown results in a lowering of the viral titer, it would be better to have a statistically significant effect to claim that it does play a proviral role. We have included one additional replicate in our analyses, but the observed reduction in viral titers remained non-significant (see Figures 2E and G). We therefore modified the message of the manuscript to rather focus on the fact that knocking down Dicer in MmNE cells does not protect against viral infection, as would be expected for an antiviral protein, but shows a tendency towards a decrease in virus accumulation. This is a notable difference compared to published data, as well as to our new data on Tadarida brasiliensis lung epithelial (Tb1Lu) cells, where a slight proviral effect of Dicer knock down can be observed (see below).*

3. a) In figure 4 and 5, the authors nicely show that mmDicer accumulate to cytoplasmic foci in M. myotis cells upon infection with SFV and SINV and these foci co-localise with double-

stranded RNA. The authors used a commercial polyclonal antibody against Dicer (A301-937A, Bethyl according to the Material and Methods section) which is specific to human Dicer to carry out their immunostaining in bat cells. The authors should provide evidence that this antibody indeed recognises/crossreacts with mmDicer as well and that the staining shown is indeed specific to mmDicer localisation especially because the heterologous expression of HA-tagged version of mmDicer in HEK 293T NoDice cells did not show this accumulation of cytoplasmic foci. The authors should verify the specificity of their mmDicer immunostaining by performing the same labelling in bat cells in which Dicer is knock-downed (or knock out) by individual and validated siRNA against mmDicer. The decrease signal of bat Dicer staining using the anti-human Dicer antibody would indicate specificity.

> *<u>Reply:</u> the reviewer is correct in its assertion and we performed experiments designed to verify the specificity of the antibody used in our immunostaining assays. Thus, we did an immunostaining of Dicer in Dicer knockdown MmNE cells and could confirm that this resulted in a decrease in signal compared to the control (Figure S4A and B).*

3. b) Another complementary approach would be to test their Dicer staining between HEK NoDice cells (no Dicer present) versus NoDice complemented with either mmDicer or human Dicer constructs, which would then indicate how much the anti-human Dicer antibody recognises bat Dicer.

> *<u>Reply:</u> We also performed an immunostaining of Dicer in HEK293 NoDice cells stably expressing either FLAG-HA tagged hDicer or mmDicer and observed that the signal for Dicer was visible in both cases, but not in control NoDice cells (Figure S4C). We measured the increase in Dicer signal upon expression of either human or bat Dicer and observed no difference between the two proteins (Figure S4D). In addition, since the constructs were tagged, we also did a FLAG immunostaining. This showed a similar localization between FLAG and Dicer, although it proved difficult to quantify the overlap since the signal was quite diffuse.*

3. c) In addition, the authors should overexpress HA-tagged mmDicer in M. myotis nasal epithelial cells and test whether HA-mmDicer accumulate into foci upon infection using an anti-HA immunostaining. This would confirm that these accumulation into foci indeed is specific to mmDicer but also would reinforce the authors' findings that host factors within bat cells are important for this formation into foci since mmDicer expression in HEK 293T No Dice cells didn't show this phenotype upon infection (figure 6). OPTIONAL: it would be interesting to overexpress HA-tagged human Dicer into M. myotis nasal epithelial cells as well to then test using anti-HA staining whether human Dicer in presence of host factors from the bat can accumulate into cytoplasmic foci or not upon viral infection.

> *<u>Reply:</u> we tried to perform the suggested experiment, but we encountered difficulties to overexpress the constructs in MmNE cells. Maybe a strong expression of Dicer in these cells is toxic or they are partially resistant to lentiviral transduction. Although we could not directly address this particular suggestion, we have performed experiments in another bat cell line (Tb1Lu) as mentioned above, and the results reinforce our hypothesis of a correlation*

between Dicer relocalization and its antiviral activity. See our reply to points 4 and 5 below.

4. This reviewer appreciates that this might be judged as beyond the scope of this study since it is focused on the role of Dicer in M. myotis. However, the observation that mmDicer accumulates into foci containing as well viral dsRNA is very interesting and it would significantly improve the manuscript if the authors would provide further indications that this phenotype is related to the lack of antiviral activity of mmDicer compared to what has been previously shown in other bat species (P.alecto and T. brasiliensis). In other words, is this accumulation of mmDicer into foci responsible for its different impact on virus infection? It would therefore be insightful to compare Dicer localisation upon infection in M. myotis versus P.alecto and/or T. brasiliensis bat cells in which Dicer was shown to be antiviral and test whether this accumulation in foci is only observed in bat cells in which Dicer is proviral (M. myotis) but not in the other bat cells in which Dicer is antiviral (P.alecto and/or T. brasiliensis).

5. OPTIONAL: Given the difference between the provial role of mmDicer compared to the antiviral activity of Dicer in cells from P.alecto and T. brasiliensis bat cells, it would strengthen the authors' findings. if additional experiments would be conducted in parallel using M. myotis, P.alecto and/or T. brasiliensis cells. Notably knocking down Dicer in both M. myotis, P.alecto and/or T. brasiliensis cells, compare the impact on viral infections with SINV, SFV, VSV and correlate any observed difference in phenotype with putative variations in the formation of foci.

<u>Reply:</u> we thank the reviewer for this very pertinent suggestion. While it proved difficult to obtain the PaKi cells from P. alecto bats, we have obtained the Tb1Lu cells from T. brasiliensis and have looked at Dicer localization in this model. In parallel, we also knocked down Dicer in these cells and looked at the impact on SINV infection. The results, shown in Figure 5 and discussed on lines 342-355, indicate that Dicer knock-down in this cell line results in a modest but significant proviral effect for one of the two siRNAs tested. Importantly, we did not observe a relocalization of Dicer upon SINV infection of Tb1Lu cells. Although this remains correlative at this stage, it reinforces the hypothesis that there is a link between Dicer localization in cytoplasmic foci and its antiviral activity.

Minor comments

• Figure 2I: The authors performed a knockdown of Dicer in M. myotis nasal epithelial cells and monitor the impact on VSV-GFP infection. They found that knocking down Dicer leads to an increase in GFP protein and RNA levels suggesting an antiviral role of Dicer while, in contrast, no effect is observed on the production of infectious particles (figure 2H). On the western blot there is only a slight/weak increase of GFP protein level observed upon Dicer knockdown. Yet, the quantification of the band intensity shows a 4-fold increase relative to tubulin and compared to cells treated with siRNA control. This 4-fold increase seems exaggerated given the low increase in the intensity shown on the blot. This discrepancy is most likely due to the lower intensity of tubulin in the western blot analysis of siDicer-treated cells compared to siNeg-treated cells. The authors should reload their western blot with equal amount of protein extract loaded to ensure that the results shown on the western blot are in line with the quantification.

Reply: we have reloaded the western blots and redone the quantification of these western blots and the results show a 2-fold increase in GFP signal upon Dicer knock-down (Figure 2I).

• Figure 3D: the authors mention that in both HEK293T cells and M. myotis nasal epithelial cells infected with SINV-GFP, there was an enrichment of 22-nucleotides (nt) paired positive and negative sense reads that overlapped with a 2-nt overhang, typical of Dicer cleavage. In Figure 3D, the data shows indeed that the duplexes are enriched for reads of 22-nt but it is unclear how this analysis reveals a 3' 2nt overhang within these duplexes. Can the authors clarify this point and if the data provided in that particular analysis indeed doesn't allow to detect these overhangs, please rephrase accordingly or provide additional analysis to support that point.

Reply: In this Figure (1D in the revised version), the graphs show the probability of pairing of all 22 nucleotides sequence mapping either to the plus or the minus strand of the viral RNA. Thus, for each sequence mapping to the plus strand, the number of sequences mapping to the minus strand with a full or partial overall is counted. A corresponding probability of pairing and Z score is calculated for each number of overlapping nucleotides (for more information on the calculation see Antoniewski (2014) Computing siRNA and piRNA Overlap Signatures. In Animal Endo-SiRNAs: Methods and Protocols, Werner A (ed) pp 135–146. New York, NY: Springer). The Z score peaks for an overlap of 20 nt in both HEK293T and M. myotis nasal epithelial cells infected with SINV. This means that there is a higher probability of two 22 nt sequence to pair along 20 nt, and thus that there are two unpaired nucleotides at the extremities of the duplexes. Initially, we also performed this analysis for VSV-infected cells, but following a comment from the editor that the number of pairs might too low to allow this (a hypothesis that we can confirm upon verifying this), we removed it from the figures. We rephrased the text in the manuscript to make this point clearer (lines 156-162).

• Typo: page 5, line 152: the authors mention that Dicer knock down had an antiviral effect against VSV-GFP infection at the RNA and protein levels. However, the data in Figure 2I and 2J show an increase in both GFP RNA and proteins levels upon knockdown of Dicer. Although this data suggests that Dicer is antiviral against VSV, the knockdown of Dicer itself is not antiviral but rather proviral/increase virus accumulation. Please rephrase this sentence to avoid confusions.

Reply: thank you for spotting this typo. We have corrected it accordingly.

Reviewer 2.

1. Figure 1 relies on transduction of cells and antibiotic selection to obtain mmDicer-expressing cells. Although we would expect that every cell expresses the construct of interest, this is not always the case, depending on the cell type and toxicity of the construct. As the constructs are tagged, I suggest that the authors use flow cytometry to measure expression levels in a single cell manner. While doing so, they can infect with SINV-GFP and correlate GFP signal with construct expression in each cell, providing a more accurate measurement of mmDicer effect

on viral infection. Alternatively, the authors could use live microscopy, as done in Fig 2, to obtain similar data.

> <u>Reply:</u> *the reviewer is correct that we did not go for monoclonal selection of our mmDicer-expressing cells and therefore that there could be some cell-to-cell variation in expression. However, we have done immunostaining of Dicer in these cells and did not see drastic differences in expression, so we do not think this should impact SINV-GFP expression in a major way (see Figure S4C). Hereafter, we provide the quantification of the Dicer signal in different conditions. Some differences in expression across cells can be observed, but they are similar between conditions and can even be observed in non-transduced cells. We have not included these quantifications in the manuscript, but would be happy to do so if the editor considers them to be important.*

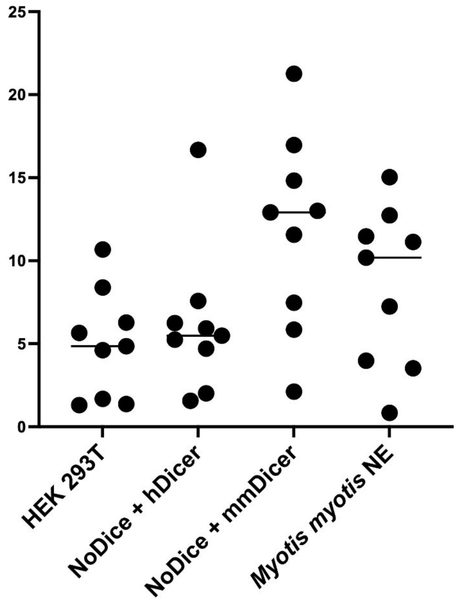

Measured intensities of Dicer immunostaining signal (arbitrary units) in several cells taken across multiple microscopy images for the indicated samples. The increased signal in expression in NoDice:mmDicer cells was also observed by western blot analysis. Overall, there appears to be indeed variations in Dicer expression between cells, but these can be observed also in non-transduced cells (HEK293T and MmNE cells) and therefore are likely not a strong contributor of the differences in phenotype between samples.

2. For Fig 1C and 1F, it would be great to have growth curves with two different MOIs, instead of a single time point, to ensure that a putative antiviral effect is not missed. Same goes for Fig 2C, especially when the authors document quite a big defect on GFP expression (a proxy for SINV infection) when Dicer is knocked down (Fig 2B). There may be a bigger difference in titers at earlier time points. This matter runs throughout the manuscript. I do not suggest that the authors should provide growth curves every time viral titers are measured, but it is still worth doing it for the 2-3 key experiments of the paper.

> <u>Reply:</u> *we have performed growth curves of virus infection for the key experiments in the manuscript as suggested. In the case of the knock-down*

experiment (original Figure 2C, now Figure 2E), the observed effect on viral titer is similar to the titer difference we obtained for MOI 0.2 (original Figure 2C, now Figure 2E) at 8h and 24h, although this difference is lower for MOI 2. As mentioned in our reply to point 2 of Reviewer 1, our results indicate that Dicer knock-down does not protect against infection, as would be expected if it played an antiviral role. Interestingly, the growth curve experiment proved more informative in the case of heterologous expression of mmDicer in HEK NoDice cells. Here, we observed an antiviral effect at every time point compared to cells expressing human Dicer (see Figure S6D) with two different MOIs. This antiviral effect was also observed at the GFP protein and viral RNA level (Figure 6D and F). In fact, in the original version of this manuscript, a small reduction in viral titers could be observed in Fig. 1C, although it was not significant. We have therefore changed the message of our manuscript, to state that when expressed in human cells, mmDicer does play a moderate antiviral role. Since in this particular setup, mmDicer does not relocalize in cytoplasmic foci, this is in agreement with our initial hypothesis of an inverse correlation between subcellular localization and antiviral activity. See text on lines 393-395 and 416-422.

3. Figure 4, could the authors provide a proof that the Dicer antibody is specific in the bat context? This can be done by staining Dicer in bat cells knocked down for Dicer and infected with SINV. The apparition of foci upon anti-Dicer antibody staining should be abbrogated or severely impaired by the knock-down.

<u>Reply:</u> see our reply to point 3 of Reviewer 1. We have not performed the analysis upon SINV infection since it might prove difficult to analyze. Indeed, we observed a reduction of virus accumulation upon Dicer knock-down, so it means that the infection might work better in cells that have not been transfected with the siRNA, thereby rendering the analysis of the pictures complicated.

4. Fig 5C, please provide a quantification of the images.

<u>Reply:</u> these microscopy images have not been quantified because they have been obtained with an epifluorescence microscope. Indeed, the Pearson correlation coefficient can only be obtained using a confocal microscope. We have used a confocal microscope to take pictures of these FISH images, but the SINV gRNA signal was weak and we had to increase the exposure time to get a proper visualization. Furthermore, there was a very large difference in signal intensity between HEK293T and M. myotis cells, making it difficult to define a signal threshold compatible for both cell lines. We nonetheless added the confocal images of this experiment in Figure S5C and show the overlay of signal for Dicer and SINV (+) RNA, which indicates a colocalization in the case of MmNE cells but not HEK293T cells.

5. l.263, when comparing this work with the recent publications on bat antiviral RNAi, the authors could also provide the percentage identity between Dicers from different species.

Reply: this is a valid point; we have looked at the percentage identity between Dicer proteins from different bat species and have included this in the revised manuscript (see Figure S7).

Reviewer 3.

1. Without direct comparison to the other bat species Dicers (especially where RNAi activity has been suggested as antiviral in previous publications) there is little in this paper that can be concluded about global aspects of bat dicer/RNAi.

Reply: see our reply to point 4 of Reviewer 1. We have performed the experiments in Tb1Lu cells and observed no relocalization of Dicer upon infection and a mild proviral effect on SINV upon its knock-down.

Minor

2. What rules out that the mmDicer re-localization observed in the immortalized mm nasal epithelial is due simply to greater expression levels over the NoDice cells heterologously expressing mmDicer?

Reply: we provide in this revised version an immunoblot showing the level of Dicer expression between HEK NoDice + mmDicer and MmNE cells (see Figure S6A). Interestingly, the level of mmDicer expression is much higher in transduced NoDice cells compared to the bat cells. We can therefore rule out a dose effect to explain the re-localization.

3. Although partially addressed in the text stating the generally long half-life of miRNAs, it seems the simplest explanation for this observation is due to some activity of a shorter-lived miRNA is required for optimal alphavirus replication is the mm nasal epithelial cells.

Reply: this is an interesting hypothesis that would prove difficult to test in a reasonable amount of time. We mentioned this possibility in the discussion of the revised manuscript (see lines 491-492).

Suggestions that could enhance the magnitude of conclusions that can be drawn from this work.

Major

• Making NoDice cells expressing other bat species Dicers, including those with claims that RNAi is antiviral, would address how universal these current observations are to bats/cell lines.

Reply: we have transduced the NoDice cells with Tadarida brasiliensis (tb) Dicer and have repeated our analysis by comparing these cells with the mmDicer-expressing cells. We have also included catalytic mutant versions (CM) of both proteins. The results, shown in Figure 6, indicate that both mmDicer and tbDicer have a moderate antiviral effect on SINV. It seems therefore that outside its natural context, mmDicer does have an antiviral phenotype, similarly to tbDicer, and that this activity might be modulated by species-specific factors that remain to be identified.

• Including an immunoblot showing that mm cells express mmDicer no more abundantly than the heterologous NoDice cells would allow ruling out the trivial explanation that foci occur at a certain critical mass of Dicer

> *Reply: yes, we have provided this piece of data as stated in reply to point 2.*

Minor
• I believe line 151 " In contrast, Dicer
knock down had an ANTIVIRAL effect against VSV-GFP infection at the RNA and protein levels, but no difference in titers was found (Fig. 2H-J)." should be " In contrast, Dicer
knock down had an PROVIRAL effect against VSV-GFP infection at the RNA and protein levels, but no difference in titers was found (Fig. 2H-J)."

> *Reply: thank you for spotting this error, which was also mentioned by Reviewer 1, we have corrected this in the text.*

Editor
I wonder whether the Z score plots of the VSV infections (in HEK and M myotis cells; Fig. 3D) are informative, given that they are based on very few overlapping reads. The authors may consider removing these plots, or alternatively, indicating in the figure the number of pairs underlying the plot.

> *Reply: we have looked into the raw data used to generate these plots and indeed the number of pairs is very low for VSV. We have therefore decided to remove these plots.*
