# Supplementary material for: Bat Dicer antiviral role and subcellular localization differ upon alphavirus infection in two distinct species
Source: PLoS Pathog. 2025 Dec 22;21(12):e1013815. doi: 10.1371/journal.ppat.1013815 (PMC12753048; doi:10.1371/journal.ppat.1013815)
Supplement: Attachment — Submitted filename: response_to_reviewers.pdf [file ppat.1013815.s010.pdf]

Response to reviewers and editor

We would like to thank the Editor for the very efficient and rapid evaluation of our revised manuscript and for acknowledging the extensive additional work that went into it. We would however like to argue that the requested experiment that he deemed would be essential before the manuscript can be accepted would significantly delay the publication of the manuscript and might not be as informative as expected.

The Editor indicates that we should perform *"(viral) small RNA profiling of infected Tb1Lu cells"*, and adds that *"The authors suggest that Dicer has antiviral activity in Tadarida brasiliensis Tb1Lu cells, which contrasts with their observation in Myotis myotis cells. It will be important to study whether these cells produce viral siRNAs as an indication that the observed antiviral effect is small RNA/RNAi dependent."*

We agree that it would indeed have been interesting to compare the viral small RNA profiles of *Myotis myotis* nasal epithelial (MmNE) cells and *Tadarida brasiliensis* lung epithelial (Tb1Lu) cells upon SINV infection. The expectation would be of course that, if RNAi is more efficient in the latter, one should detect an increased accumulation of viral siRNAs compared to the former. However, the accumulation of small RNAs resembling siRNAs in a total RNA extract does not necessarily indicate that these are functional and active against the virus. Indeed, looking at our small RNA sequencing data in SINV-infected HEK293T cells (shown in Figure 1), one would expect that these cells are indeed mounting an RNAi-based response since we detect an siRNA-like signature in the viral reads. However, we know that this is not the case since we show in Figure 6 (D-F) that there is no difference in terms of virus accumulation between HEK-NoDice cells complemented with an empty vector of a vector expressing the human protein. Similarly, Tsai et al. reported in 2018 that human Dicer can produce Influenza A virus derived siRNA in the absence of the NS1 protein, but that these siRNAs had almost no inhibitory effect due to their inefficient loading into Argonaute proteins (Tsai et al. RNA 2018 Sep;24(9):1172-1182. doi: 10.1261/rna.066332.118).
The only direct evidence that could indicate a contribution of the RNAi machinery to antiviral defense is the impact of knocking-down or out essential factors in the RNAi machinery (i.e. Dicer or Ago2). We show that knocking-down Dicer in Tb1Lu cells results in an increase of Sindbis virus accumulation (Figure 5), while it has the opposite effect in MmNE cells (Figure 2). In addition, we also show that expressing either Myotis myotis Dicer or Tadarida brasiliensis Dicer in HEK NoDice cells results in a decrease of virus accumulation (Figure 6). Whether this is indeed due to a direct RNAi-like type of response in HEK293T cells also remains to be formally proven. Actually, we could also perform small RNA sequencing in these bat Dicer expressing human cells to go along the small RNA sequencing of Tb1Lu cells. Nonetheless, we respectfully think that this would go beyond the scope of this paper and would require embarking on a whole new set of experiments to have all the proper controls in place and to make sure they are efficiently loaded into Argonaute proteins. This being said, our data suggest that the two bat Dicers do not behave exactly the same if we look at the effect of expressing the catalytic dead mutants on viral titer accumulation (Figure 6E).

We modified the discussion of the manuscript to take all these considerations into account and to justify the absence of small RNA sequencing data in the Tb1Lu cell line. The added text can be found on pages 20 and 21, lines 487 to 510.

We hope that with these changes the Editor will reconsider his decision regarding the addition of new experimental data in our manuscript.
